# DNA-Based Optical Quantification of Ion Transport across Giant Vesicles

**DOI:** 10.1021/acsnano.2c07496

**Published:** 2022-10-12

**Authors:** Marcus Fletcher, Jinbo Zhu, Roger Rubio-Sánchez, Sarah E Sandler, Kareem Al Nahas, Lorenzo Di Michele, Ulrich F Keyser, Ran Tivony

**Affiliations:** † Cavendish Laboratory, University of Cambridge, J.J. Thomson Avenue, CambridgeCB3 0HE, U.K.; ‡ Department of Chemistry, Molecular Sciences Research Hub, Imperial College London, LondonW12 0BZ, U.K.; § fabriCELL, Molecular Sciences Research Hub, Imperial College London, LondonW12 0BZ, U.K.

**Keywords:** ion transport, ion channels, giant unilamellar vesicles, microfluidics, G-quadruplex, ion sensor

## Abstract

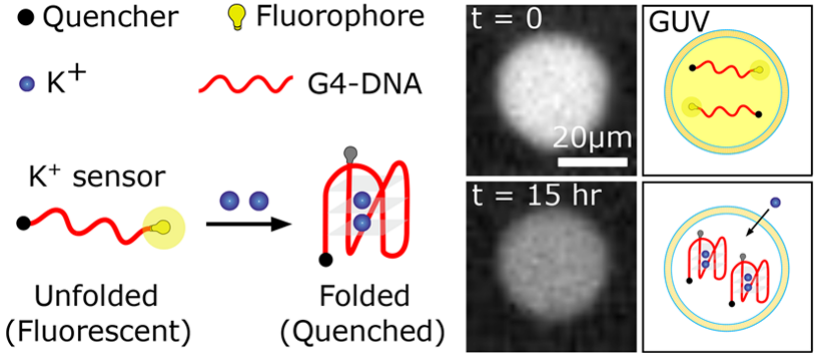

Accurate measurements of ion permeability through cellular membranes remains challenging due to the lack of suitable ion-selective probes. Here we use giant unilamellar vesicles (GUVs) as membrane models for the direct visualization of mass translocation at the single-vesicle level. Ion transport is indicated with a fluorescently adjustable DNA-based sensor that accurately detects sub-millimolar variations in K^+^ concentration. In combination with microfluidics, we employed our DNA-based K^+^ sensor for extraction of the permeation coefficient of potassium ions. We measured K^+^ permeability coefficients at least 1 order of magnitude larger than previously reported values from bulk experiments and show that permeation rates across the lipid bilayer increase in the presence of octanol. In addition, an analysis of the K^+^ flux in different concentration gradients allows us to estimate the complementary H^+^ flux that dissipates the charge imbalance across the GUV membrane. Subsequently, we show that our sensor can quantify the K^+^ transport across prototypical cation-selective ion channels, gramicidin A and OmpF, revealing their relative H^+^/K^+^ selectivity. Our results show that gramicidin A is much more selective to protons than OmpF with a H^+^/K^+^ permeability ratio of ∼10^4^.

The movement of ions across the lipid membrane is a fundamental process in a cell’s endeavor to maintain homeostasis. Mediated by an array of transport proteins, such as ion pumps and ion channels, ionic flows are harnessed by cells and organelles to induce electrochemical gradients that power vital biochemical activities from nutrient uptake^1,2^ to energy conversion.^3^ At the same time, however, ions may directly diffuse through the lipid bilayer, acting to dissipate the induced gradient. Therefore, the necessary coupling between multiple translocation pathways for the precise regulation of ionic fluxes in cells makes permeability measurements in living systems highly challenging.

Giant unilamellar vesicles (GUVs), micrometer-sized compartments enclosed by a lipid bilayer, offer a simplified biomimetic membrane model for studying ion transport phenomena at the single-vesicle level and under tightly controlled conditions and membrane compositions.^4,5^ Moreover, with dimensions and membrane composition comparable to those of cells, GUVs find applications as protocells and can accommodate different types of biological modules to possess cell-like features.^6−9^ Hence, studying ion transport in GUV systems is not only important for the mere interest of resolving transport mechanisms but also for elucidating the energetics of protein machines such as pumps and motors. However, while methods for developing increasingly realistic artificial cell models using GUVs are rapidly growing in number, techniques to investigate ion transport are scarce.^4,5,10^ Predominantly, GUVs are analyzed using fluorescence microscopy approaches. Therefore, one of the main reasons for the lack of ion transport studies in GUV systems is the absence of suitable fluorescent probes for sensing physiologically important ions with high sensitivity and selectivity.

Ion-responsive dyes are particularly suited to measurements of ion transport in GUVs, as they can be readily encapsulated within the GUV lumen.^4,10,11^ Therefore, by combining microfluidic approaches such as hydrodynamic trapping with real-time fluorescent imaging, large populations of GUVs can be monitored in parallel and at the single-vesicle level,^12−15^ enabling the study of one vesicle at a time,^5^ unlike the case for whole-GUV patch-clamping. Nevertheless, only a small number of ion-responsive dyes are available commercially and suitable for membrane transport measurements. For instance, pyranine (or HPTS), a pH-sensitive dye, is a well-established fluorescent probe for quantifying proton transport in liposomes,^16−21^ and in some cases, it has also been used to indirectly evaluate K^+^ transport rates from monitoring pH changes.^22^ However, for other ions such as K^+^, the most abundant cation in the intracellular fluid, there is only one commercially available fluorescence indicator, potassium binding benzofuran isophthalate (PBFI).^23^ Still, since PBFI has relatively low sensitivity to potassium (*K*_d_ = 9–70 mM),^24^ it has traditionally been used for monitoring the intracellular level of K^+^ under physiological electrolyte concentrations^25^ and has not been routinely adopted to direct measurements of K^+^ transport in GUVs. Therefore, although potassium ions play an important role in cellular processes such as action potential induction and osmotic pressure regulation, as far as we are aware, no measurements of potassium ion transport across GUV systems have been reported to date. For these reasons, we sought to develop a fluorescent-based probe suitable for measuring K^+^ transport in GUV-based synthetic cells.

It is well-known that certain G-rich sequences of single-stranded DNA (ssDNA) fold to G-quadruplex secondary structures (G4) in the presence of specific cations,^26−28^ especially potassium ions.^29^ While the concept of using G4-based sensors for K^+^ has already been demonstrated,^30,31^ their application in K^+^ transport studies has not been investigated thus far.

Here we employed a G4-based potassium ion sensor using a human telomere G4 sequence labeled with a fluorophore (FAM) and a quencher (Q) at its two ends (i.e., FAMQ-G4). Upon folding of the telomere G4 sequence, the fluorescence intensity of FAM decreases significantly following its interaction with the quencher. We show that FAMQ-G4 can readily detect sub-millimolar variations in K^+^ concentration and be used for a quantitative analysis of potassium transport in GUVs. Furthermore, through modifying the fluorophore at one end of our DNA-based sensor, we were able to customize the wavelengths at which K^+^ is detected, demonstrating its potential use in combination with other fluorescent probes in multi-ion transport studies. By utilizing microfluidic-based techniques to prepare and trap GUVs with encapsulated FAMQ-G4, we directly quantified the permeation coefficient of K^+^ across the lipid bilayer of GUVs at the single-vesicle level. Taking the same analysis approach, we measured the transport rate of K^+^ through membranes containing two well-characterized membrane channels, gramicidin A and OmpF, and evaluated the effect of each type of channel on the buildup of transmembrane potential based on their H^+^/K^+^ selectivity and intrinsic permeation pathways.

## Results and Discussion

### Design of a DNA-Based K^+^ Sensor for Transport Measurements

**Figure 1:**
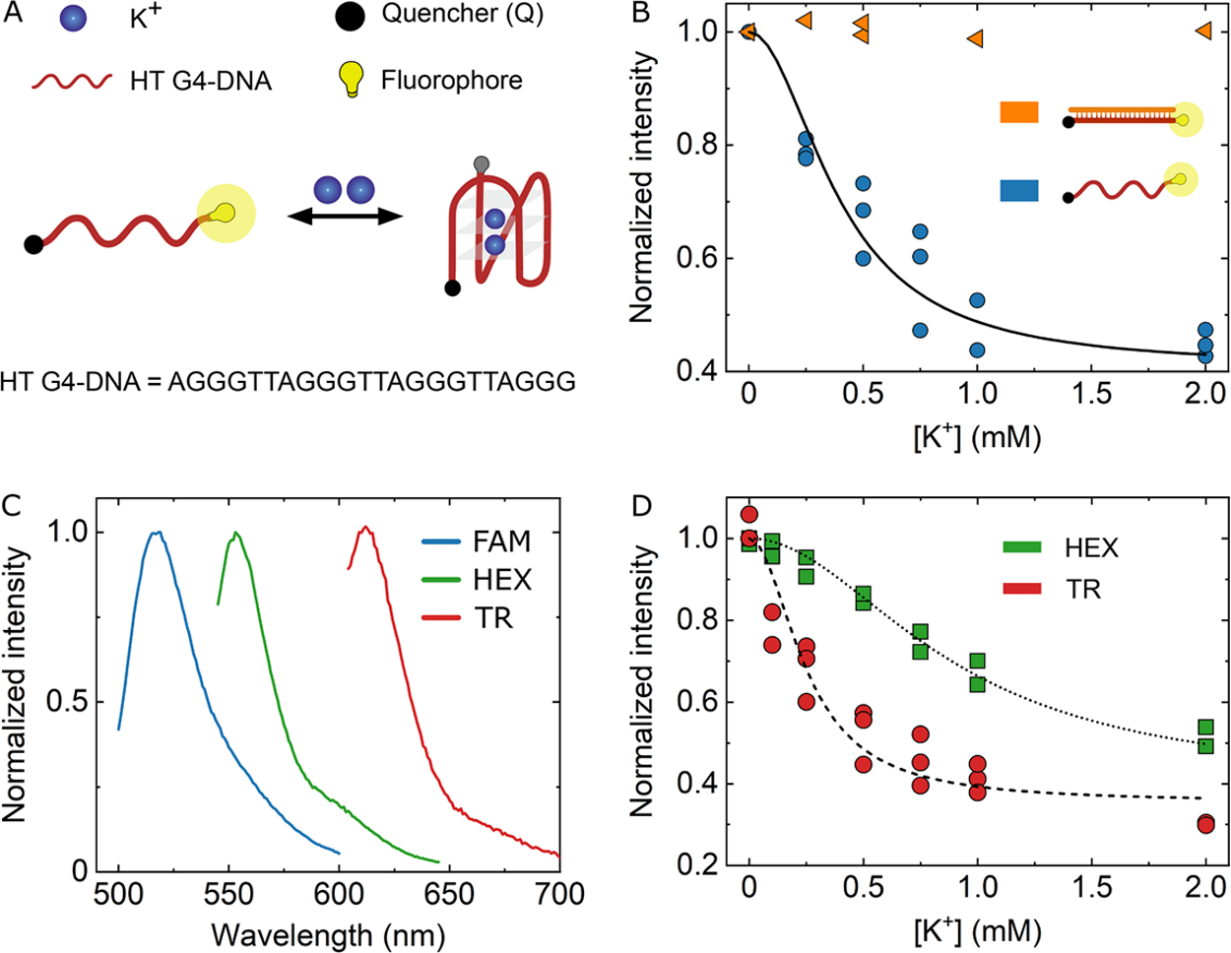
Design and characterization of G-quadruplex (G4) DNA based K+ probes. (A) Schematic illustrating the K^+^ sensing principle of G4-DNA probes. A human telomeric DNA (HT G4-DNA), modified with a fluorophore and quencher at opposing ends (5′ and 3′, respectively), folds in response to K^+^, thereby bringing the fluorophore and quencher into closer contact and decreasing the fluorescence intensity of the probe. (B) Variation of FAMQ-G4 fluorescence intensity with increasing concentration of K^+^ (blue circles). In the presence of a complementary DNA strand (orange triangles) no reduction in fluorescence is observed for the double-stranded G4-DNA, indicating that folding of the single-stranded G4-DNA in the presence of K^+^ causes the fluorescent response. (C) Emission spectra of G4 probes modified with different fluorophores at their 5′ end. (D) Fluorescence intensity variation of G4 probes, modified with HEX (green) or Texas Red (red), in response to increasing K^+^ concentration.

In our quest to design a suitable ion-responsive sensor for transport studies we have considered the following basic guiding principles: (1) high ion sensitivity, (2) no photobleaching during the time scale of the experiment, (3) negligible permeation through the lipid bilayer, and (4) minimal disruption to membrane properties. DNA nanotechnology offers powerful design techniques for self-assembly of nanostructures with many sensing applications.^31^ Ueyama et al. showed that a guanine (G)-rich oligonucleotide (Human Telomere G4-DNA) can be used as an efficient potassium sensor in aqueous media.^32^ To detect the presence of K^+^, G4-DNA was labeled at one end with a FRET donor and at the other end with an acceptor. Thus, upon interaction with potassium it folds to a tetraplex structure, known as G-quadruplex (G4), and generates a FRET signal. Since then, similar concepts have been followed and G4-based sensors have been widely employed for various biosensing applications.^33,34^ Nevertheless, the use of G4-based sensors in membrane transport studies has not been demonstrated thus far. We chose the Human Telomere G4-DNA sequence AGGG(TTAGGG)_3_ (Figure 1A), which retains high specificity for K^+^ ions and folds into a hybrid type G-quadruplex in the presence of K^+^.^29,35^ To enable the fluorescence detection of potassium, we modified the G4-DNA by attaching a fluorophore (fluorescein, 6-FAM) to its 5′ -end and a quencher (Iowa black FQ, Q) to its 3′-end (Figure 1A). As schematically illustrated in Figure 1A, upon binding two potassium ions, the obtained G4-DNA sensor (FAMQ-G4) folds to a stable tetraplex structure and the distance between the fluorophore and quencher decreases. As a result, the fluorescence intensity drops significantly and plateaus at 0.45 when [K^+^] > 1 mM (Figure 1B), implying a sensing region of 0 < [K+] < 1 mM for FAMQ-G4.

To quantify the sensitivity of FAMQ-G4 to potassium ions, we investigated the probe’s fluorescent response at different K^+^ concentrations. Figure 1B shows that the relative fluorescence of FAMQ-G4 (blue circles) gradually decreases with increasing concentrations of K^+^, indicating that more G-quadruplex structures form when a larger amount of K^+^ is available to interact with FAMQ-G4. We verified that the fluorescence intensity reduction is due to folding of the G4 probe and is not a result of direct interaction between potassium ions and FAM by first mixing FAMQ-G4 with its complementary sequence (CCCTAA)_3_CCCT, to form a more rigid double-stranded DNA (dsDNA) structure, and then measuring the fluorescence at increased potassium concentration. As depicted in Figure 1B (orange triangles), no detectable change in fluorescence was observed, confirming that the measured fluorescence reduction is solely due to the folding of FAMQ-G4. By fitting the relevant binding model (Supporting Information) to the fluorescence data (blue line), we evaluated the dissociation constant between potassium and FAMQ-G4 to be , where [K^+^] is the concentration of potassium ions, and [G4_uf_] and [G4_f_] are the concentrations of FAMQ-G4 in its unfolded and folded configurations, respectively. The obtained *K*_d_ agrees well with a previously reported dissociation constant measured for a similar G4 structure using mass spectrometry,^26^ indicating the high affinity between K^+^ and FAMQ-G4. Correspondingly, we were able to detect sub-millimolar variations in potassium ion concentration (Figure 1B), unlike the case for the only commercially available potassium ion indicator PBFI, which responds to concentration changes in the millimolar range (*K*_d_(PBFI) is between 9 and 70 mM).^24,36^ Notably, another benefit of our G-quadruplex-based sensor over conventional ion-sensitive dyes is the ability to tune its affinity to potassium (i.e., *K*_d_) through changing its sequence.^37^ In addition, we found that FAMQ-G4 also folds in response to Na^+^, though with lower sensitivity relative to K^+^ (Figure S1). Therefore, even in the presence of 5 mM Na^+^, FAMQ-G4 retains sensitivity to K^+^ (Figure S2). This result, however, does not necessarily suggest that our G4-DNA sensor may be suitable for quantifying potassium concentrations in living cells, where sodium concentrations are much higher (100–150 mM). Still, we note that in systems where the type and concentration of electrolytes can be carefully chosen and controlled, for instance by using Li^+^ instead of K^+^ or Na^+^ (Figure S3), FAMQ-G4 can be suitably used to detect either sodium or potassium, respectively.

Another important advantage of DNA-based fluorescent probes over conventional molecular dyes is their customizability. Typically, in ion transport studies, the permeation of the examined ion is coupled to the simultaneous flow of other types of ions. Therefore, to understand the combined influence of different ions in processes, such as electrochemical gradient development or the activity of transporters, it is of interest to determine the flux of several types of ions at the same time. We show that FAMQ-G4 can be adjusted using different types of fluorophores and quenchers to enable detection of K^+^ at various excitation/emission wavelengths while retaining high sensitivity. Figure 1C shows the excitation/emission spectra of three G4-DNA-based designs, FAMQ-G4, HEXQ-G4, and TRQ′-G4, where Q′, HEX, and TR stand for Iowa Black-RQ, hexachlorofluorescein, and Texas Red, respectively. Importantly, regardless of the attachment type, all of the aforementioned DNA probes retain sub-millimolar sensitivity to changes in potassium concentration with comparable *K*_d_(HEXQ-G4) = (7.8 ± 0.2) × 10^–7^ M^2^ and *K*_d_(TRQ′-G4) = (6.3 ± 2.0) × 10^–7^ M^2^ (Figure 1D and the Supporting Information).

### Potassium Ion Permeation across Single GUVs

**Figure 2:**
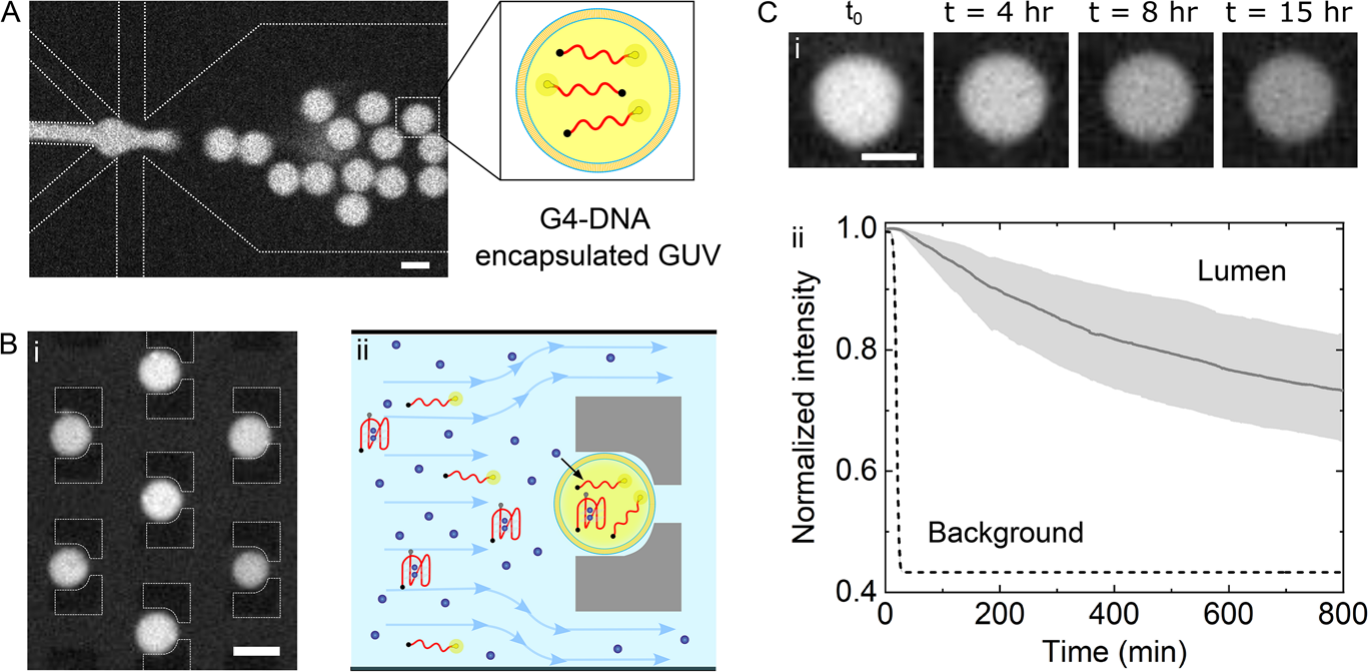
K^+^ transport measurements across single GUVs using microfluidic-based approaches. (A) On-chip production of GUVs (DOPC/DOPG, 3:1 w/w) and encapsulation of FAMQ-G4 (10 μM), using octanol-assisted liposome assembly. Scale bar: 20 μm. (B) FAMQ-G4 encapsulated GUV immobilization using microfluidic hydrodynamic trapping (i). Once trapped, a 1 mM KCl solution, containing 500 nM FAMQ-G4, is perfused into the microfluidic chamber (ii), where GUVs can be visualized for >10 h, allowing one to capture the transport process of slow-permeating solutes such as K^+^. Scale bar: 40 μm. (C) (i) Time lapse of lumenal GUV fluorescence during the K^+^ transport process, showing the decrease in FAMQ-G4 fluorescence as a result of K^+^ induced folding. Scale bar: 20 μm. (ii) Analysis of K^+^ permeation across the lipid bilayer of single GUVs (*n* = 441) showing the temporal variation of lumenal (black solid line) and background (black dashed line) fluorescence intensity (median). The gray bands represent the lower and upper quartiles of the measured fluorescence at each time point for each measured vesicle.

To confirm that our DNA-based sensor is suitable for ion transport studies *in vitro*, we utilized FAMQ-G4 to quantify the permeation of K^+^ across the lipid bilayer of giant unilamellar vesicles (GUVs). For this purpose, we used a droplet-based microfluidic approach, octanol-assisted liposome assembly (OLA), to encapsulate FAMQ-G4 (10 μM), dissolved in buffer A (10 mM Tris, 100 mM sucrose buffer, pH = 7.6), in the interior of negatively charged DOPC/DOPG GUVs (Figure 2A and the Supporting Information). The formed GUVs were then extracted and perfused into a separate microfluidic device, where they were immobilized using hydrodynamic trapping (Figure 2B(i)). We note that the FAMQ-G4 concentration inside the GUVs shows some polydispersity between different experiments, most likely due to extraction of GUVs from the OLA production device to the trapping device. Nevertheless, once trapped, all GUV intensities were stable before perfusion of KCl and no leakage of the DNA fluorescent probe was observed (Figure S4). We account for the polydispersity in fluorescence intensity by normalizing it according to the intensity prior to the transport of K^+^. In addition, since the quenching time of FAM (∼10 s), due to folding of our DNA sensor following the addition of 100 μM KCl, was found to be comparable to the rate of data acquisition in our experiments (Figure S5), the sensitivity of FAMQ-G4 to K^+^ influx should not be affected by its folding kinetics.

As illustrated in Figure 2B(ii), to initiate the permeation of potassium ions across the GUV membrane, we perfused 1 mM KCl solution (also consisting of 500 nM FAMQ-G4 in buffer A) into the microfluidic chamber and monitored the intensity of FAMQ-G4, outside and inside the GUVs, over time. By trapping GUVs in a microfluidic platform, we were able to precisely control the flow rate, monitor the arrival of potassium ions to the trapped GUVs, and analyze their flux across single vesicles over long periods—an essential time frame for determining the membrane transport of ions with very low permeation rates (Figure 2C(i)). As such, we were able to monitor the arrival of K^+^ precisely and accurately. In addition, no leakage of FAMQ-G4 through the GUV lipid bilayer or photobleaching were observed during the time scale of our experiments (Figure S4). We also verified, using conductance measurements, that FAMQ-G4 does not disrupt the lipid bilayer integrity or form pores that may render the GUV membrane more permeable to ions (Figure S6).

**Figure 3:**
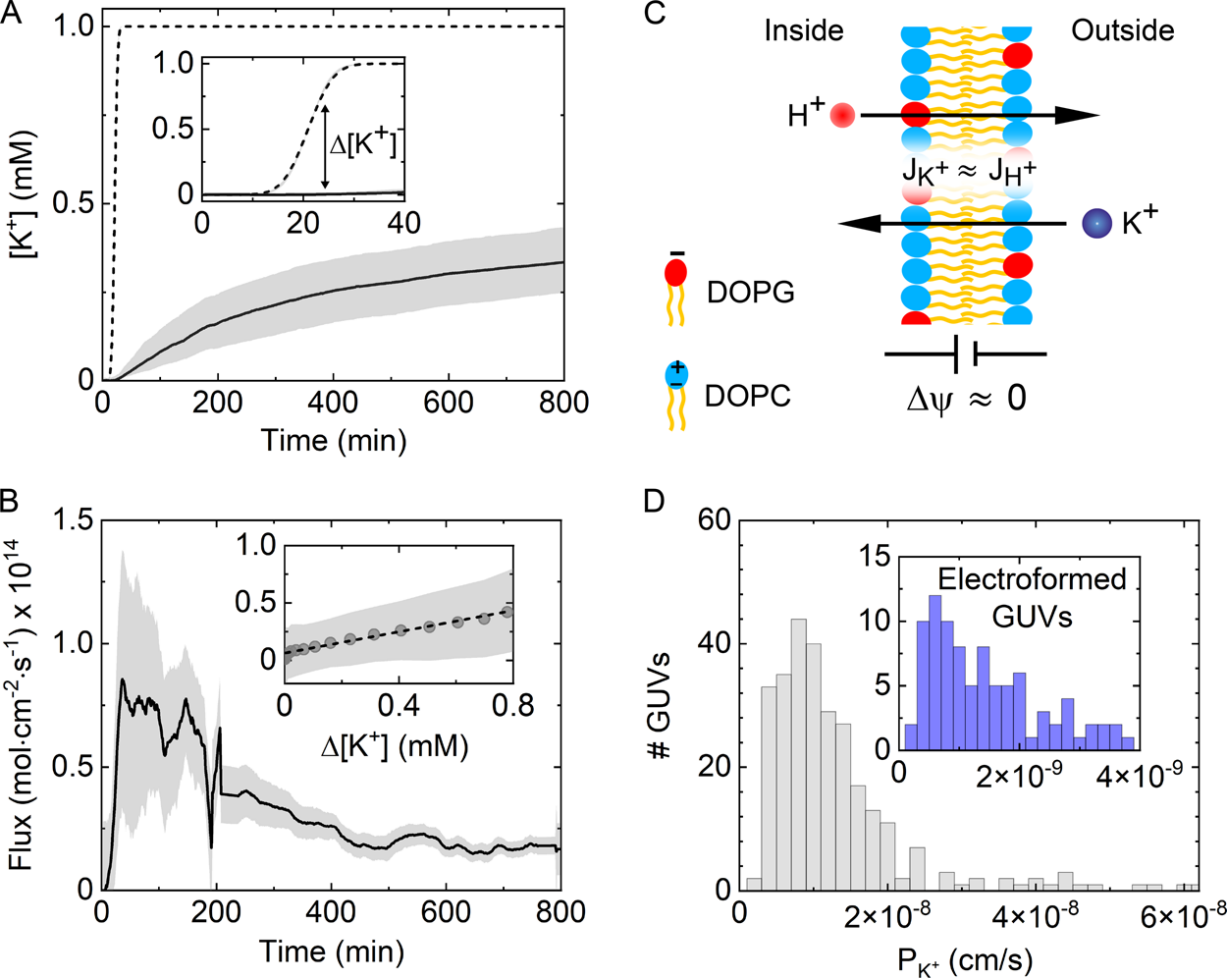
Quantification of K^+^ permeability across single GUVs. (A) Variation of K^+^ concentration inside (solid) and outside (dashed) GUVs during the transport process. The distribution of [K^+^] among GUVs is represented by the upper and lower quartiles of [K^+^] at each time point. Inset: magnified view of the transmembrane K^+^ concentration gradient, Δ[K^+^], generated in the initial period (typically a few tens of minutes) of our experiments. (B) Variation of K^+^ flux into GUVs over the measurement time course. Inset: flux profile showing the variation of K^+^ flux as a function of Δ[K^+^] during the initial period of Δ[K^+^] development. The obtained linear flux profile can be represented through *J* = *P*Δ[K^+^], thus enabling the determination of K^+^ permeability from the slope of the curve. (C) Schematic showing the proposed dissipation mechanism of transmembrane potential by a counter flux of protons across the GUV lipid bilayer in our experiments. (D) Distribution of measured permeability coefficients for negatively charged OLA DOPC/DOPG (3:1) GUVs (*N* = 441). Inset: measured permeability distribution of electroformed DOPC/DOPG (3:1) GUVs.

By analyzing the measured time-dependent fluorescence intensities of FAMQ-G4 inside and outside the trapped vesicles (Figure 2C(ii)), we resolved the sub-millimolar variation of lumenal and extravesicular potassium concentrations, [K^+^]_i_ (solid curve) and [K^+^]_o_ (dashed curve) respectively, and determined the temporal evolution of potassium concentration gradients across the GUV membrane over the course of more than 13 h following the arrival of K^+^ (Figure 3A). Consequently, we observed that the gradual increase of [K^+^]_i_ commences only after [K^+^]_o_ reached its maximum value, indicating the establishment of a potassium concentration gradient of Δ[K^+^] = [K^+^]_o_ – [K^+^]_i_ ≈ 1 mM prior to the movement of K^+^ across the GUV membrane (inset to Figure 3A). Furthermore, the significantly slower increase of K^+^ concentration within the GUVs shows that the rate of potassium permeation is not limited by diffusion through the unstirred layer. Importantly, we note that the ability to investigate the transport of K^+^ under small concentration gradients (i.e., Δ[K^+^] ≤ 1 mM) ensures that flux measurements are carried out under low osmotic differences and, thus, with minimal perturbation of the GUV membrane.^38,39^

Next, we determined the influx density of potassium from the obtained temporal evolution of [K^+^]_i_ through *J*_K^+^_ = d[K^+^]_i_/d*t* × *r*/3, where *r* is the radius of each detected GUV (Figure 3B). We note that our direct measurement of GUV dimensions at the single-vesicle level allows us to reduce inaccuracies caused by assumptions of radii as in bulk experiments. Under our experimental conditions, the influx of K^+^ is determined by two opposing driving forces, the concentration difference (Δ[K^+^]) and transmembrane potential (Δψ) across the membrane, as described by the Goldman–Hodgkin–Katz (GHK) flux equation^20,40^
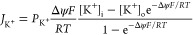
where [K^+^]_i_ and [K^+^]_o_ are the concentrations of K^+^ (mol/cm^3^) inside and outside the GUV, respectively, *P*_K^+^_ (cm/s) is the permeability coefficient of K^+^, and *F*, *R*, and *T* have their usual meanings. However, in cases where Δψ ≈ 0, the flux density is expected to change linearly with concentration gradient according to Fick’s first law:
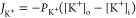


By plotting the potassium influx as a function of Δ[K^+^], we found that at the early period of transport (10–20 min) the flux profile *J*_K^+^_(Δ[K^+^])) across single GUVs changes in a rather linear fashion (inset to Figure 3B), suggesting a negligible development of Δψ during this time frame. One way to justify the insignificant development of Δψ is by considering its dissipation by fluxes of other ions with permeability rates higher than those of potassium, as schematically shown in Figure 3C. For instance, protons (H^+^) are known to cross the lipid bilayer at rates ∼10^7^ times higher than those of other monovalent ions such as K^+^.^10,21^ In a previous study, we reported that the permeation coefficient of protons across the same composition of OLA-GUVs is *P*_H^+^_ ≈ 0.002 cm/s.^10^ Since the concentration gradient of protons during the early period of K^+^ permeation (i.e., at the linear regime) is negligible and [H^+^]_o_ ≈ [H^+^]_i_ (Figure S7), it can be shown that even under a very low transmembrane potential of Δψ = 1 mV the estimated efflux of protons at pH = 7.6 is comparable to that of potassium (inset to Figure 3B). We estimated *J*_H^+^_ = *P*_H^+^_[H^+^](*F*Δψ/(*RT*)) = 2 × 10^–15^ mol cm^–2^ s^–1^ according to the GHK flux equation, where [H^+^] = 10^–pH^/1000 is the concentration of protons on both sides of the membrane (mol/cm^3^).

Such a near 1/1 stoichiometry of the net K^+^ and H^+^ fluxes (Figure 3C), which was also observed across small unilamellar vesicles,^23^ enables the determination of the potassium permeability coefficient directly from the linear regime of the flux profile using *P*_K^+^_ = *J*_K^+^_/Δ[K^+^]. Figure 3D shows the measured permeability coefficient distribution of potassium permeation coefficients with a mean value of *P̅*_*K*^+^_ = (1.5 ± 0.2) × 10^–8^ cm s^–1^. Notably, the obtained *P*_K^+^_ values are several orders of magnitude higher in comparison with previously reported K^+^ permeabilities, which typically fall in the range between 10^–10^ and 10^–12^ cm s^–1^.^16,23^ To account for this discrepancy, we considered the possibility that trace amounts of octanol may reside in the lipid bilayer of OLA GUVs, thus rendering it more permeable to cations relative to oil-free membranes.^41^ To this end, we encapsulated our DNA-based probe in electroformed GUVs with a lipid composition similar to that of OLA GUVs and measured the permeability of K^+^ under the same experimental conditions (Figure S8). As shown in the inset to Figure 3D, the obtained potassium permeability coefficients, in the absence of octanol, were found to be 1 order of magnitude lower than in the case of OLA GUVs, with an average value of *P̅*_*K*^+^_ = (1.3 ± 1.5) × 10^–9^ cm s^–1^, suggesting that residual octanol molecules are indeed incorporated in the membrane of OLA GUVs.

An additional contribution to the relatively high measured *P*_K^+^_ values is the negative surface potential of the lipid bilayer. In the case of protons, for instance, a 2-fold increase in permeation coefficient was measured for a negatively charged lipid bilayer (DOPC/DOPG), with a composition similar to that in this study, compared to an undercharged membrane (DOPC).^10^ Likewise, Koyanagi et al. compared ion transport rates across small unilamellar vesicles comprising either POPC or POPC/POPG (1/1) lipids and observed a factor of 3 increase in transport rates with negatively charged lipids present.^42^ We note, however, that while previously attained *P*_K^+^_ values may still be somewhat lower, the present study signifies a model-free analysis of K^+^ permeation at the single-vesicle level, unlike earlier bulk studies that measured average *P*_K^+^_ values using an ensemble of nanometric liposomes.

### Potassium Transport and H^+^/K^+^ Selectivity of Gramicidin A and OmpF

In living cells, the passive movement of ions across the membrane and down the electrochemical gradient is primarily catalyzed by ion-selective channels that discriminate ions mostly based on the atomic composition and size of their binding site.^43^ For instance, gramicidin A (gA) is a short peptide (15 amino acids) that forms a cation-selective bilayer-spanning channel with pore dimensions that restrict ions and water to move in single file through it.^44^ As a result, protons (H^+^), which can rapidly hop along the water wire inside the pore, permeate through gA at much higher rates than other monovalent cations such as K^+^ and Na^+17^. On the other hand, other cation-selective channels, such as the Gram-negative trimeric porin OmpF (outer membrane protein F), enable higher K^+^ permeation^45−47^ which may approach that of H^+^. Therefore, it is of interest to evaluate the relative H^+^/K^+^ selectivity (i.e., the permeability ratio) of these two prototypical channels and understand how these two distinct channels may affect the development, or dissipation, of the transmembrane potential.

We studied the passive transport of K^+^ through gA and OmpF using the same basic principles shown in Figure 2. To incorporate the channel proteins (either gA or OmpF) in the lipid bilayer of OLA-GUVs, we perfused the protein solution into the trapping chamber containing the GUVs and then washed away any residual proteins (see Materials and Methods). Importantly, following the incorporation stage we verified that no leakage of FAMQ-G4 occurs through the channels during the period of our measurements (Figure S9). In addition, we note that in each gA experiment we observed two populations of GUVs with different transport rates (Figure S10)—one population with rates comparable to those of OLA GUVs without gA and the other with significantly greater decay rates. Similarly, the occurrence of two populations was also found in the case of OmpF, though to a lesser extent (Figure S11). The observed heterogeneous incorporation of functional gA, which has also been widely observed for different membrane active peptides and proteins,^12,48,49^ clearly signifies the advantage of single-vesicle transport measurement over bulk experiments in which contributions from both populations are averaged. Here, for clarity, we removed the slow population of GUVs from further analysis. Furthermore, to verify the successful incorporation of gA into the GUV membrane, we performed the K^+^ transport experiments following perfusion of different gA concentrations ([gA] = 0.5 or 5 ng/mL) and found that transport rates are [gA] dependent, indicating successful channel insertions (Figure S10).

**Figure 4:**
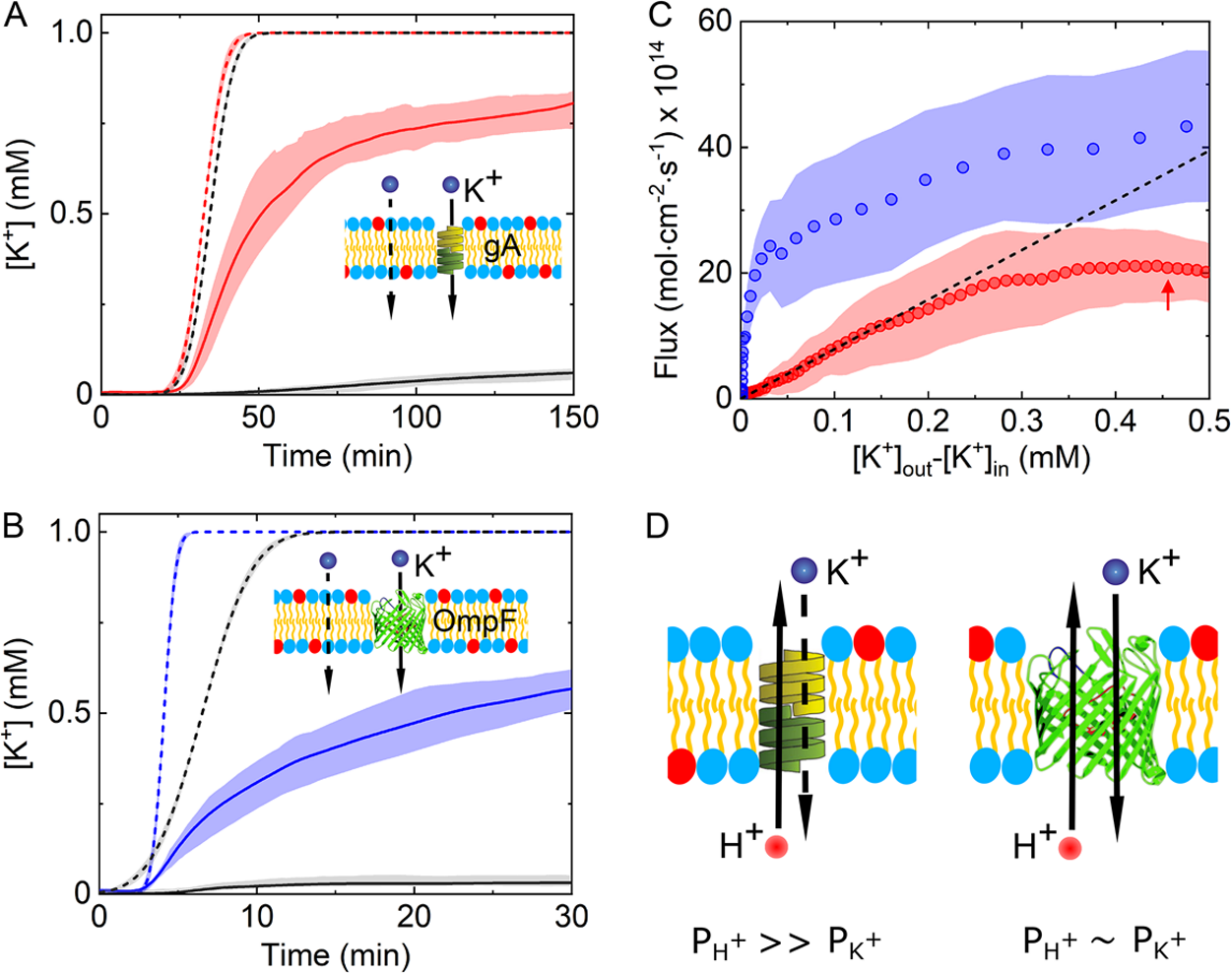
K^+^ transport kinetics across GUVs with reconstituted model ion channels. (A) Time-resolved variation of lumenal (solid lines) and extravesicular (dashed lines) K^+^ concentration for DOPC/DOPG (3:1) GUVs with (red, *n* = 46), and without (black, *n* = 38) reconstituted gramicidin A (gA) (see experimental section). The distribution of permeated [K^+^] over GUVs is represented by the upper and lower quartiles of [K^+^] at each time point. Inset: schematic illustrating the two possible transport pathways across gA incorporated GUVs. **B.** Analysis of lumenal (solid lines) and extravesicular (dashed lines) [K^+^] across DOPC/DOPG (3:1) GUVs with (blue, *n* = 83) and without (black, *n* = 76) reconstituted OmpF (see Materials and Methods). Inset: schematic illustrating the two possible transport pathways across OmpF-incorporated GUVs. (C) Flux profiles obtained for GUVs with reconstituted gA (red) and OmpF (blue). The circles are the mean flux values, and the bands are the lower and upper quartiles for each GUV population. The black dashed line is the best fit of a linear curve to the mean flux data at the linear regime (0 < Δ[*K*^+^] < 0.13 mM), using linear regression. The red arrow indicates the Δ[K^+^] value at which the measured mean flux (red circles) is 0.58 of the flux (black dashed line) at the same Δ[K^+^] in the absence of transmembrane potential development. (D) Schematic demonstrating the suggested origin for the variance in H^+^/K^+^ selectivity between gA and OmpF.

Figure 4 shows the temporal variation of lumenal and extravesicular potassium concentrations across GUVs containing either gA (Figure 4A, red curves) or OmpF (Figure 4B, blue curves). For comparison, we show the variation of [K^+^] in a GUV system without incorporated proteins (black curves), measured under the same conditions (see also Figures S10 and S11). As can be seen, the presence of gA and OmpF in the GUV membrane dramatically increases the transport rate of K^+^ relative to the plain lipid bilayer. We stress, however, that under our experimental conditions K^+^ movement across the GUV membrane may occur through the lipid bilayer and ion channels at the same time (see insets to Figure 4). Therefore, the membrane permeability coefficient in our system should encompass the contribution from both pathways: *P*_K^+^_^m^ = ϕ*P*_K^+^_^ch^ + (1 – ϕ)*P*_K^+^_^lb^, where ϕ and 1 – ϕ are the membrane area fractions occupied by reconstituted channel proteins and the lipid bilayer, respectively, and *P*_K^+^_^ch^ and *P*_K^+^_^lb^ are their corresponding permeability rates. Nevertheless, since it can be clearly seen from our measured data in Figure 4 that *P*_K^+^_^m^ ≫ *P*_K^+^_^lb^ when gA and OmpF are incorporated in the lipid bilayer, the overall permeability rate of K^+^ through all reconstituted ion channels in a GUV () can be estimated from the measured membrane permeability as . Likewise, the measured flux density of potassium ions across the membrane is determined by passive transport across the ion channels so that *J*_K^+^_^m^ ≈ *J*_K^+^_^ch^.

By comparing the obtained flux profiles *J*_K^+^_^m^(Δ[K^+^]) for gA and OmpF at early stages of transport (Figure 4C), i.e. once the flux increase starts to saturate as Δ[K^+^] increases, it can be seen that in the presence of gA the flux profile deviates from linearity at Δ[K^+^] ≈ 0.16 mM (or 1.6 × 10^–7^ mol/cm^3^), while no such deviation could be detected for OmpF. The absence of a detectable linear regime in the measured OmpF flux profiles implies that the deviation from linearity occurs at very low Δ[K^+^] values and is faster than the data acquisition rate used in these experiments (6.6 frames/min). Still, the earlier deviation and development of Δψ due to K^+^ influx across OmpF indicates that compensation of charge imbalance by H^+^ efflux occurs at much slower rates than in the case of gA. Hence, by considering that Cl^–^ flux through cation-selective channels is negligible,^44,47^ the observed variation in flux profiles strongly suggests that the H^+^/K^+^ selectivity of gA is higher compared to OmpF.

In the absence of a linear regime in the case of OmpF, we evaluated only the H^+^/K^+^ selectivity of gA by estimating the ratio between the overall permeabilities of K^+^ and H^+^ across it (). To obtain , we consider that the influx of K^+^ through gA and efflux of H^+^ across both gA and the lipid bilayer are kept approximately similar in the early stage of K^+^ transport when Δψ < RT/F so that *J*_K^+^_^gA^ ≈ *J*_H^+^_^gA^ + *J*_H^+^_^lb^ (Supporting Information). Also, recalling that [H^+^]_o_ ≈ [H^+^]_i_ ≈ 10^–7.6^/1000 mol/cm^3^ during the same initial transport period (Supporting Information),  can be related to the flux of K^+^ according to . Thus, when *F*Δψ/*RT* = 1 (or, alternatively, when Δψ = 25 mV at 25 °C), the channel permeability to protons can be approximated through . According to the GHK flux equation, the flux of potassium is expected to drop by ca. 42% relative to its value in the absence of transmembrane potential. By taking  cm/s^10^ and evaluating *J*_K^+^_^gA^ (Δ[K^+^], Δψ = 25 mV) from the measured flux profile (red arrow in Figure 4C), we found that  cm/s. Additionally, from the linear regime of the gA flux curve we obtained  cm/s (black dashed line in Figure 4C). Taken together, the evaluated H^+^/K^+^ permeability ratio for gA is , as similarly reported elsewhere.^17^ The obtained H^+^/K^+^ selectivity value, while approximated, clearly reflects the intrinsic characteristics of the gA pore that contains a single water wire along which protons can “hop” without displacing water molecules as other cations such as K^+^.^50^ For that reason, gA was also observed to effectively dissipate the buildup of Δψ across the GUV membrane. In the case of OmpF, however, the fast generation of charge imbalance at very low K^+^ gradients clearly indicates that it possesses a much lower H^+^/K^+^ selectivity value relative to gA. In addition, the comparable conductances of different cations across OmpF^51^ suggest that the permeability rates of K^+^ and H^+^ are similar as well (Figure 4D). Overall, as illustrated in Figure 4D, our results suggest that σ_gA_ ≫ σ_OmpF_ due to the significantly larger H^+^/K^+^ permeability ratio of gA relative to OmpF.

## Conclusions

To summarize, we utilized a transmembrane ion transport sensing technique by combining a G-quadruplex-forming fluorescent ion sensor with microfluidic GUV handling techniques. We showed that the designed G-quadruplex DNA probe detects sub-millimolar changes in the lumenal concentration of K^+^ and can be customized to provide a fluorescent signal over a range of wavelengths. By quantifying the fluxes and concentration gradient of potassium ions across single GUVs, we were able to determine the permeation rate of K^+^ across the lipid bilayer and estimate the complementary H^+^ fluxes that dissipate the transmembrane potential during the initial stages of K^+^ transport. In addition, we showed that the presence of oil residues, such as octanol in our case, significantly increases the permeability rate of potassium through the lipid bilayer by 1 order of magnitude. Still, even in the absence of octanol in the membrane of GUVs, the measured K^+^ permeability coefficients were found to be at least 1 order of magnitude larger than permeability values that were previously obtained from bulk experiments. Employing the same approach, we measured the passive transport of K^+^ across two archetypical cation-selective ion channels, gramicidin A and OmpF, incorporated in the membrane of GUVs. An analysis of potassium fluxes across the GUV membrane in the presence of these two channel types provided a useful insight into their role in determining the rate of charge accumulation inside the vesicles. Considering that these channels are cation selective, we evaluated the H^+^/K^+^ selectivity of gramicidin A and showed that it is much more selective to protons than OmpF. We anticipate that our study will guide future measurements of ion transport across relevant biological systems such as ion channels, ion pumps, and ionophores.

## Materials and Methods

### Materials

1,2-Dioleoyl-*sn*-glycero-3-phosphocholine (DOPC), 1,2-dioleoyl-*sn*-glycero-3-phospho-(1-ac-glycerol) (sodium salt; DOPG), and 1,2-dioleoyl-*sn*-glycero-3-phosphoethanolamine-*N*-(lissamine rhodamine B sulfonyl) (ammonium salt; 18/1 Liss Rhod PE) were purchased from Avanti Polar Lipids as powders and dissolved in ethanol to final concentrations of 100 mg/mL (DOPC and DOPG) and 0.5 mg/mL (Liss Rhod PE). Fluorophore- and quencher-modified DNA strands (Figure 1A) were synthesized and purified (HPLC) by Integrated DNA Technologies (IDT) and received at 100 μM in IDT TE Buffer pH 8. 1-Octanol was purchased from Sigma and used as received. Polydimethylsiloxane (PDMS; Sylgard 184) was purchased from Dow Corning and used as received. Potassium chloride (KCl) powder was purchased from Sigma and dissolved in a buffer (10 mM Tris, 1 mM EDTA) to a concentration of 1 M. Gramicidin A was purchased as a powder from Sigma and immediately dissolved in ethanol (analytical grade) at 5 mg/mL and stored at 4 °C. Outer membrane protein F (OmpF, 5.5 mg/mL) in a 1% octyl-POE solution was kindly provided by Prof. Mathias Winterhalter and stored at 4 °C.

### Fluorescence Examination of the G4-DNA K^+^ Sensor

Human Telomere G4-DNA (AGGG(TTAGGG)_3_), modified with a fluorophore (FAM, HEX, or Texas Red) at its 5′-end and a quencher (Iowa Black FQ for FAM and HEX and Iowa Black RQ for Texas Red) at the 3′-end, were slowly brought to room temperature from their storage at −20 °C and diluted to 100 nM with a 10 mM Tris 1 mM EDTA buffer. To study the response of G4-DNA to K^+^, different concentrations of KCl, varying from 0 to 2 mM, were added to the probe solution. For preparation of the dsDNA, both FAMQ-G4 and its complementary strand were heated to 60 °C for 10 min and then mixed in a 1/1 volume ratio, where the volume of each sample was 300 μL. The fluorescence emission spectra were recorded from 505 to 605 nm at an excitation of 488 nm for FAM, from 545 to 645 nm for HEX at an excitation of 535 nm, and from 575 to 675 nm at an excitation of 559 nm for Texas Red, using a Cary Eclipse fluorescence spectrophotometer (Agilent Technologies, USA). Spectra at each K^+^ concentration were analyzed using a custom-made Python program. The total fluorescence intensity for each sample was calculated by numerically integrating the spectrum over the range of wavelengths. The relative fluorescence change was then determined by normalizing the fluorescence at each K^+^ concentration by the fluorescence with no added K^+^ for each experiment.

### Microfluidic Chip Fabrication

Master molds for the microfluidic designs were created by photolithography and soft lithography. A layer of SU-8 2025 (Microchem) was deposited (3800 rpm, 60 s with 100 rpm/s acceleration for the initial 38 s) onto a 4 in. silicon substrate (University Wafers, USA). Then, the wafer was prebaked (2 min at 65 °C, 5 min at 95 °C) and selectively exposed to ultraviolet light (12 s, 365–405 nm, 20 mW cm^–2^) using a direct laser writer (LPKF ProtoLaser LDI, Germany). The sample was postbaked (2 min at 65 °C, 5 min at 95 °C), developed for 3 min, dried with a gentle stream of nitrogen, and hard baked for 15 min at 125 °C. To create a negative replica from the silicon mold, we used standard soft lithography techniques. Sylgard 184 polydimethylsiloxane (PDMS) was mixed with a curing agent (9/1 w/w, Dow Corning), and the mixture was poured onto the silicon mold and baked at 60 °C for 55 min. The PDMS chip was then peeled off the mold and inlet/outlet columns created using a 0.75 mm biopsy punch (Miltex). The chip was plasma-bonded to a PDMS-coated glass coverslip using an air plasma (10 W, 25 sccm, 10 s exposure, Diener Electronic GmbH & Co. KG, Germany). The OLA formation device was further processed with a PVA coating of the post junction channel, as previously described in detail elsewhere.^52^ The microfluidic chip (Figure S12) was plasma-treated for 1 min immediately before device operation.

### Microfluidic Experimental Design

Two microfluidic devices were used in this work, one for vesicle formation using the octanol-assisted liposome assembly (OLA) device and another for hydrodynamic trapping of GUVs. The production of GUVs using the OLA has been previously described in detail elsewhere^52−54^ and is also elaborated in the Supporting Information. Briefly, OLA produces GUVs at a six-way junction at which three different liquid phases meet, the inner aqueous (IA), lipid-octanol (LO), and outer aqueous (OA). The LO and OA channels both bifurcate from their inlets to create the five flows that meet at the formation junction (Figure S12). By tuning the flow rates of these three phases, double-emulsion (IA/LO/OA) droplets are produced. The octanol spontaneously phase separates, producing a lipid vesicle (GUV) and an octanol droplet.

The trapping device consists of two fully independent networks, each containing four chambers of trap arrays. The trapping principle has been described in previous reports.^52^ Separation of the formation and trapping devices into two isolated chips enables simpler user operation of both devices and the ability to remove GUVs from the microfluidics and store with little added operation time. CAD designs for both microfluidic devices are available in the Supporting Information and can be seen in Figure S12.

### Microfluidic Device Operation

All microfluidic devices were operated and imaged on an Olympus IX73 inverted microscope. In- and out-of-plane motion was controlled automatically by a motorized XY stage (Prior Scientific Instruments Ltd. UK). Applied pressures were controlled via a pressure-driven pump (MFCS-EZ, Fluigent GmbH, Germany) and were tuned as required using the MAESFLOW software. Four pressure channels were used for the integrated vesicle-formation and filtering device (Figure S12A), while two channels were required to operate the vesicle-trapping device (Figure S12B). All input fluids were connected from their reservoirs (Micrewtube 0.5 or 1.5 mL, Simport) to the microfluidic devices through polymer tubing (Tygon microbore tubing 0.020 in. ID, 0.060 in. OD, Cole-Parmer, UK) and connector tips (isolated from dispensing tips, Gauge 23 blunt end, Intertronics). Camera acquisition was achieved using a Photometrics Evolve 512 delta camera controlled via the open-source software Micro-Manager 1.4, with magnification via a 10× air objective (Olympus UPLFLN).^55^ For fluorescence experiments, illumination was supplied by a wLS LED lamp (Q-Imaging) and passed through a FITC filter cube (Chroma).

To immobilize the GUVs during transport experiments, we trapped the vesicles in a microfluidic chip using hydrodynamic traps. The trapping device (Figure S12B) was bonded immediately prior to operation, and the OA solution was then perfused through it to avoid formation of air bubbles. Subsequently, the GUVs were introduced into the device and trapping was stopped once a satisfactory number of vesicles occupied the traps. Before the potassium solution was introduced to the GUVs, the chambers were washed with IA solution for 30 min to wash away residuals such as oil droplets, free protein (gramicidin A or OmpF), etc. In the gramicidin A and OmpF experiments the model ion channel solution (either 0.5 or 5 ng/mL gramicidin A or 0.0125 mg/mL OmpF in 10 mM Tris 1 mM EDTA, pH 7.6) was perfused into one of the inlets (each inlet was connected to four separated chambers), to enable a control measurement in parallel with the transport experiment. IA solution with 500 nM FAMQ-G4 was perfused to the trap chamber to determine the external concentration of potassium as it arrived into the trapping network. In the final perfusion step a 1 mM KCl solution (with 500 nM FAMQ-G4) was introduced to the chamber to initialize the transport of potassium. Data acquisition started prior to the final perfusion step.

### Electroformation of Vesicles

Vesicles prepared via electroformation were obtained using a protocol modified from refs (56 and 57). Briefly, indium tin oxide (ITO) slides were cleaned with 15 min sonication cycles of isopropanol followed by Milli-Q water and subsequently dried under a nitrogen flow. An ITO slide was heated to ∼50 °C, and 45 μL of lipid mixture (4 mg/mL) was gently spread on the conductive side of the slide using a glass coverslip. The slide was placed in a dry silica desiccator and kept under vacuum for 1 h. Electroformation chambers, assembled by coupling ITO slides with an ∼1 mm thick polydimethylsiloxane (PDMS) spacer, were filled with approximately 250 μL of DNA-containing solution (10 μM) rehydration buffer. The chambers were connected with clamps to a frequency generator and subjected to a sinusoidal alternating current with a voltage amplitude of 2 V. Electroformation was carried out using a frequency of 10 Hz for 2 h followed by 1 h at 2 Hz. Finally, vesicles were gently retrieved and stored at room temperature in the dark to prevent photobleaching and photo-oxidation. The electroformed GUVs were diluted 5× in IA and perfused into the hydrodynamic trapping device. To account for multilamellar GUVs, images of all experimental ROIs were taken before and after the ion transport experiment using a filter isolating the membrane fluorescence signal (Liss-Rhodamine-PE). We visually identified subpopulation vesicles at different levels of membrane fluorescence and removed all vesicles that did not belong to the lowest level of fluorescence.

### Data Acquisition

The automated stage supporting the microfluidic device and light source was synchronized with the camera exposure being triggered through Micro-Manager 1.4 software.^55^ The stage cycled through a number of fields of view, and an image was captured at each. The cycle repeated at user-specified time intervals (9 s for OmpF, 15 s for gramicidin A, and 30 or 60 s for the GUV experiment). The number of positions was chosen according to the number of points of interest in each experiment. The exposure was set to 30 ms in all experiments to avoid bleaching of the fluorescent sensor (Figure S4). The image sequences were stored by field of view into an ImageJ-compatible TIFF Stacks format.

### Image Processing

The time-lapse videos were individually processed using an in-house Python program described in detail previously.^14^ The program first identified the arrival of the potassium. As the potassium arrived, the background fluorescence smoothly varied over a few video frames to its final background intensity. The time point at which the background reached half its maximum intensity was used to identify the GUVs for measurement. The software automatically identified individual bright objects above a threshold. Because the GUVs moved slightly within their traps (±5 pixels), the software redetected each initially detected GUV center at every time point. The mean intensity within a 4 × 4 pixel region about the GUV center was recorded for the remaining video frames in the experiment. At the same time, the area of each GUV was measured using the Watershed algorithm.^58^ The background intensity for each video was also determined by calculating the median intensity over the full field of view. These intensity time series among all experimental videos were collated for further analysis. Filtering was applied to remove GUVs that suddenly escaped their traps, and those below a certain size threshold (<6 μm), which are likely burst and re-formed membranes.

## Supporting Information Available

GUV formation and filtering, Human Telomere G-quadruplex K^+^ binding model, Na^+^ response of FAMQ-G4, sensitivity of FAMQ-G4 to K^+^ in the presence of Na^+^, response of FAMQ-G4 to K^+^ in 100 mM LiCl, FAMQ-HT photobleaching and leakage across the lipid bilayer, folding kinetics of FAMQ-HT following 100 μM KCl, interaction of FAMQ-G4 with the lipid bilayer, change of pH inside GUVs during K^+^ permeation, measurement of K^+^ permeation across electroformed GUVs, demonstration of no leakage of FAMQ-G4 from GUVs after perfusion of model ion channels, efficiency of gramicidin A incorporation dependent on its concentration in solution, K^+^ transport across OmpF containing GUVs, microfluidic device CAD designs, K^+^ flux density approximately equaling H^+^ counter flux during the linear regime (PDF)
