# Supplementary material for: DNA-Based Optical Quantification of Ion Transport across Giant Vesicles
Source: ACS Nano. 2022 Oct 12;16(10):17128–38. doi: 10.1021/acsnano.2c07496 (PMC9620405; doi:10.1021/acsnano.2c07496)
Supplement: Supplementary file 1 — nn2c07496_si_001.pdf [file nn2c07496_si_001.pdf]

Supplementary information for:

DNA-based optical quantification of ion transport across giant vesicles

Marcus Fletcher[†], Jinbo Zhu[†], Roger Rubio-Sánchez[‡§], Sarah E Sandler[†], Kareem Al Nahas[†], Lorenzo Di Michele[‡§], Ulrich F Keyser[†], and Ran Tivony[†]*,

† Cavendish Laboratory, University of Cambridge, J.J. Thomson Avenue, Cambridge CB3 0HE, UK

‡ Department of Chemistry, Molecular Sciences Research Hub, Imperial College London, London W12 0BZ, UK

§ fabriCELL, Molecular Sciences Research Hub, Imperial College London, London W12 0BZ, UK

[*] Corresponding author: E-mail: rt497@cam.ac.uk

Table of contents

S1. GUVs formation and filtering..2

S2. Human Telomere G-quadruplex K+ binding model..3

S3. Na^+ response of FAMQ-G4..3

S4. Sensitivity of FAMQ-G4 to K^+ in the presence of Na^+..4

S5. Response of FAMQ-G4 to K^+ in 100mM LiCl..4

S6. FAMQ-HT photobleaching and leakage across the lipid bilayer..5

S7. Folding kinetics of FAMQ-HT following 100 µM KCl..5

S8. Interaction of FAMQ-G4 with the lipid-bilayer..6

S9. Change of pH inside GUVs during K^+ permeation..7

S10. Measurement of K^+ permeation across electroformed GUVs..8

S11. FAMQ-G4 does not leak from GUVs after perfusion of model ion channels..9

S12. Efficiency of gramicidin A incorporation depends on its concentration in solution..10

S13. K^+ transport across OmpF containing GUVs..11

S14. Microfluidic device CAD Designs..12

S15. K^+ flux density approximately equals H^+ counter flux during linear regime..12

S1. GUVs formation and filtering

OLA devices allow the preparation of GUVs with a narrow size distribution at a production rate of 10s Hz. The essential operation has been described in detail elsewhere[1]. Briefly, the production of OLA GUVs relies on the initial formation of W/O/W double emulsion droplets, then the spontaneous phase separation of the oil phase (octanol and lipids) to create a lipid membrane and an octanol droplet (which subsequently buds off under shear forces). This process is achieved by the user adjustment of three fluid flows (Inner Aqueous (IA), Lipid-Octanol phase (LO) and Outer Aqueous (OA)), each driven by separate applied pressures, meeting at a 6 way junction. The inlets to both the LO and OA phases bifurcate to create the necessary 5 channels input to the vesicle formation junction (the remaining post-junction channel serves as an outlet). This is illustrated in figure S6A. After formation, the GUVs suspension flows to an integrated filtering module (figure S6A) described previously[1]. This enables the removal of unwanted residual components produced during the formation stage, including octanol droplets. Filtering is achieved by one further input fluid flow, the washing solution, WS, meeting the output stream of the OLA formation at a Y junction following which the flow is split into 5 outlets (i.e., fractions). When the WS phase is driven at a flow rate 2-3 times larger relative to the OLA flow, the OLA mixture (i.e. octanol droplets, lipid aggregates, free dye, etc.), excluding the GUVs, is directed to fractions I and II. Conversely, the GUVs, which are only weakly deformable in the Y junction, flow to outlet III and subsequently into 0.5mL reservoirs. Each reservoir was used directly as an input to the trapping device. The formation was continued until a minimum of 200µL of GUV solution was collected. The aqueous solutions (IA, OA and WS) consisted of 100mM sucrose, 10mM Tris and 1mM EDTA in miliQ water. The base solution was further titrated with a 32% HCl solution to reach a pH = 7.6. In addition, the OA phase also contained 50mg/ml of Kolliphor P-188 (Sigma-Aldrich,UK). The FAMQ-G4 sensor was added to the IA to a concentration of 10µM. The OA and the WS were diluted with TE1 buffer until their osmolarity matched the IA to within 1 mOsm as measured by a osmometer (Roebling, Camlab). The LO phase contained a lipid composition (DOPC:DOPG:LissRhod-PE 3:1:0.04) at 6mg/ml in 1-octanol.

S2. Human Telomere G-quadruplex K+ binding model

We used the following binding model to fit the dependence of our G4-DNA probes on [K$^+$]: $I_{probe} = \frac{1-c}{\frac{[K^+]^2}{K_d}+1} + c$, where K_d is the binding constant for the 2K$^+$ + G4 ⇄ G4·(K$^+$)$_2$ binding process, and [K$^+$] is the free concentration of K$^+$ at equilibrium. K_d was used as a fitting parameter. As the concentration of G4 is significantly less than the added [K$^+$] in our measurements ([G4] = 500nM), we can approximate [K$^+$] as the concentration of potassium added. This means that for the regime, [G4] ≪ [K$^+$], the normalised response of G4 is approximately dependent on the concentration of added K$^+$ and not on [G4]. The model parameter c in this equation is the fluorescence in an excess of K$^+$ which may be extracted directly from the plateau region for each experiment (see main text).

S3. Na$^+$ response of FAMQ-G4

We investigated the fluorescent response of FAMQ-G4 to Na$^+$ following the addition of different sodium concentrations, and compared it to the response of FAMQ-G4 to K$^+$.

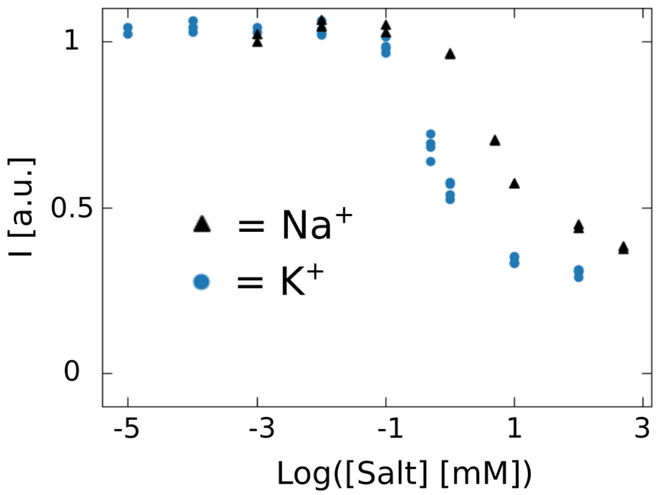

Figure S1. Comparison of FAMQ-G4 response to Na$^+$ (black triangles) and K$^+$ (blue circles) following the addition of NaCl and KCl at different concentrations.

S4. Sensitivity of FAMQ-G4 to K^+ in the presence of Na^+

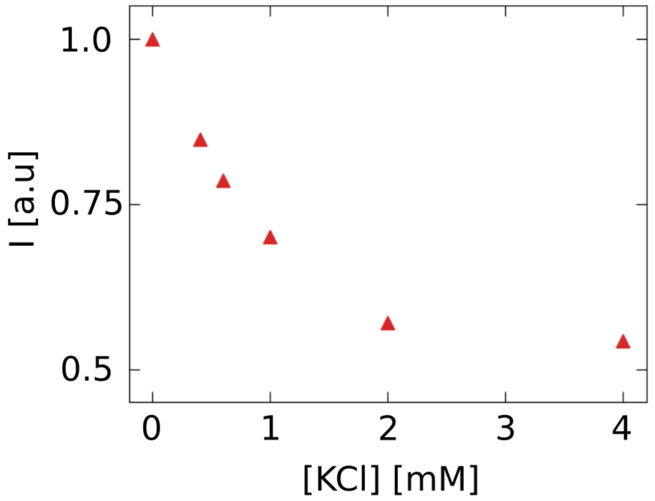

Figure S2. Normalized fluorescence intensity of FAMQ-G4 in response to various concentrations of K^+, in the presence of 5mM NaCl.

S5. Response of FAMQ-G4 to K^+ in 100mM LiCl

In cases where the response of our G4 probes to Na^+ negates the use of physiological sodium concentrations in the background solution, one may use Li^+ which is known to have very low binding to Human Telomere G4s[2]. We investigated the response of FAMQ-G4 in the presence of 100mM LiCl as shown in figure S4. As can be seen, the significant reduction of fluorescence intensity between $0 < [K^+] < 1$mM, relative to absence of Li^+, indicates that FAMQ-G4 may still be used to probe $[K^+]$ in the presence of 100mM LiCl.

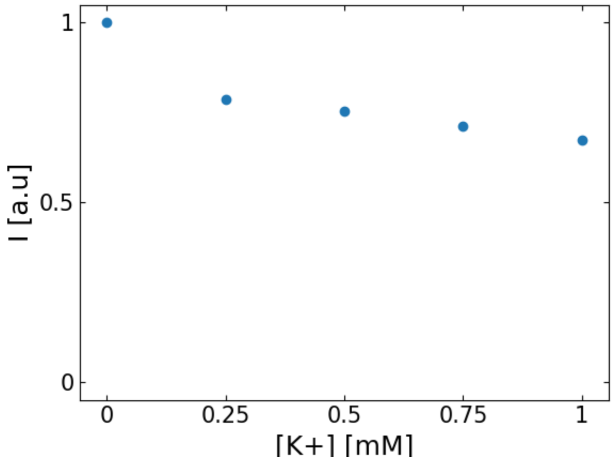

Figure S3. K^+ response of FAMQ-G4 in the presence 100mM LiCl.

S6. FAMQ-HT photobleaching and leakage across the lipid bilayer

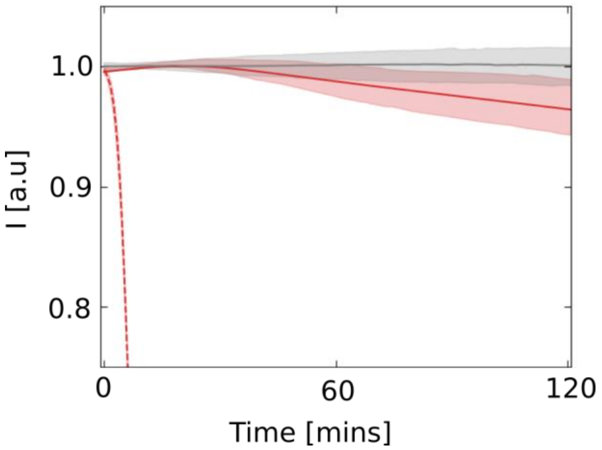

Figure S4. Photostability and membrane impermeability control of FAMQ-G4. The distribution of lumenal fluorescence within GUVs was compared with (red) and without (grey) perfusion of 1mM K^+. The arrival of K^+ is indicated by the background fluorescence decrease (red, dashed). The stability of the fluorescence without K^+ arrival indicates that the dye does not permeate the membrane, nor does it photobleach during the period of measurement.

S7. Folding kinetics of FAMQ-HT following 100 µM KCl

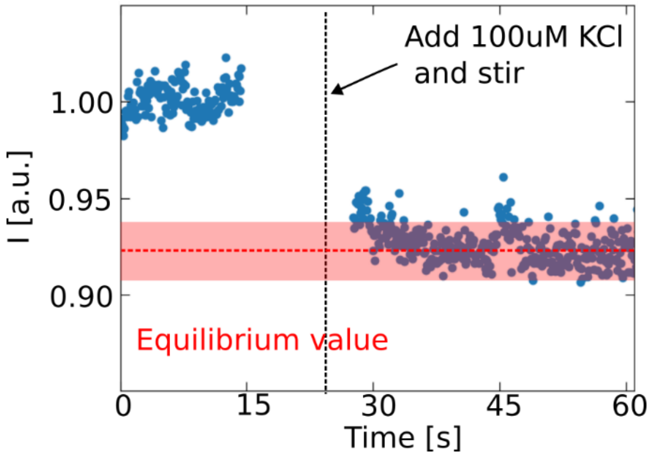

Figure S5. Kinetics of FAMQ-HT (10 µM) folding following the addition of 100 µM KCl indicating that the G4 structure is stable after approximately 10 s . The fluorescence intensity is normalized by the initial fluorescence. There is a down time of ≈10 s as the measurement is paused and a 1 µL droplet of 10mM KCl is added to the 100 µL solution of FAMQ-HT in the cuvette. The estimated time of mixing is indicated by the dashed line (black). The red line indicates the estimated equilibrium value, and the shaded region the estimated error.

S8. Interaction of FAMQ-G4 with the lipid-bilayer

To rule out any increase in ionic permeability due to interactions between FAMQ-G4 and the lipid membrane, we performed ionic conductance measurements using the black lipid membrane (BLM) method (figure S7). The current (I) of K^+ was measured across a negatively charged planar lipid bilayer (DOPC:DOPG, 3:1 w/w) having the same lipid composition as of the GUVs in the transport experiments reported in the main paper. To examine whether our DNA sensor interacts with the GUV membrane and consequently increases its permeability to K^+, we compared the conductance of the BLM with and without the presence of FAMQ-G4 (1 μM in the *cis* side). Figure S7 shows the obtained IV curves across the BLM with (red) and without (black) FAMQ-G4, where the slope of the curves represents the conductance across the membrane. As can be seen, the membrane conductance remained unaltered in the presence of FAMQ-G4, indicating that the DNA sensor does not interact with the negatively charged lipid bilayer in a way that induces pores or disrupts the integrity of the membrane. Therefore, no increase of the GUV membrane permeability to potassium ions is expected to occur in our transport experiments.

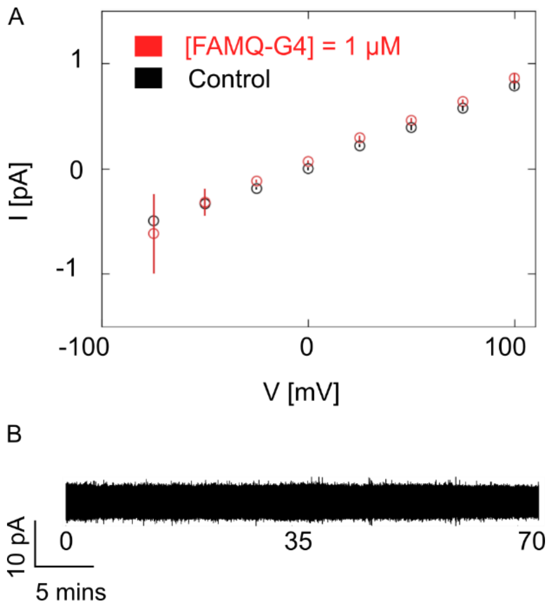

Figure S6. A. Conductance measurement of K^+ across a black lipid membrane (BLM) model. The current (I) of K^+ was measured at various voltages (V) across a negatively charged planar lipid bilayer (DOPC:DOPG, 3:1 w/w) with (red) and without (black) FAMQ-G4 (1 μM) in the *cis* side of the membrane. The experiments was performed as follows. A Teflon cuvette with a thin foil separating two chambers was used. First, both sides of a hole (Ø = 0.15 mm) in the foil which divides *cis* and *trans* were coated with hexadecane (1% in pentane). This was

allowed to incubate for 5 minutes and then 700 µL of 0.5 M KCl, 25 mM HEPES (4- (2-hydroxyethyl)-1-piperazineethanesulfonic acid), pH 7.0 was added to each chamber. Next, 5 µl of 5 mg/ml DOPC:DOPG 3:1 in pentane were added to each chamber. The solution was gently pipetted up and down in both chambers until the membrane was formed. After membrane formation, the DNA sensor was added to the *cis* side at a final concentration of 1 µM and IV curves were recorded. **B.** Ionic current vs. time across the BLM under 50 mV voltage with FAMQ-G4. The current data was acquired for 70 minutes at a sampling rate of 5 kHz using Axopatch 200B amplifier. Clampex and Clampfit softwares were used to gather and analyse the data.

S9. Change of pH inside GUVs during K^+ permeation

The presence of pH buffer in our experiment solutions allows H^+ to export in response to K^+ influx without significant change in the pH (and therefore $[\mathrm{H}^+]_{\mathrm{free}}$). To estimate the change in pH due to export of H^+ from a GUV, we determined the buffer capacity β of our buffer (100mM Sucrose, 10mM Tris, 1mM EDTA pH = 7.6) about the expected range of pH during the K^+ transport process. We titrated the buffer with known concentrations of $\Delta[\mathrm{H}^+]$ and measured the resultant pH change ΔpH. Accordingly, the buffer capacity, β, was calculated as a function of pH through $\beta(\mathrm{pH}) = \frac{\Delta[H^+]}{\Delta\mathrm{pH}}$. As can be seen in figure S4A, at our experimental pH (7.6) we measured $\beta{\sim}5\ mM$. Our measured value of β indicates that to compensate the maximum translocated amount of $[K^+]$ = 1mM with $[\mathrm{H}^+]_{\mathrm{export}}$ = 1mM, leads to a maximal theoretical increase of pH by, $\Delta\mathrm{pH} = 0.2$. Therefore, in the initial linear regime of K^+ influx where the translocated amount is $[K^+]$ ~ 100 μM, the pH change must be $\Delta\mathrm{pH} \ll 0.02$, so $[\mathrm{H}^+]_{export}$ is expected to be negligible. To confirm that pH changes insignificantly during K^+ transport we repeated the K^+ transport experiment after perfusion of gramicidin A (5ng/ml), however this time encapsulating the pH probe HPTS instead of FAMQ-G4. We maintained FAMQ-G4 (500nM) outside of the GUVs to detect K^+ arrival. Figure S5B shows the insignificant difference between GUVs exposed (blue) and unexposed (orange) to K^+, indicating that pH does not vary within GUVs during K^+ transport and that in our experiments $[\mathrm{H}^+]_{\mathrm{o}} \approx [\mathrm{H}^+]_{\mathrm{i}}$ during potassium transport.

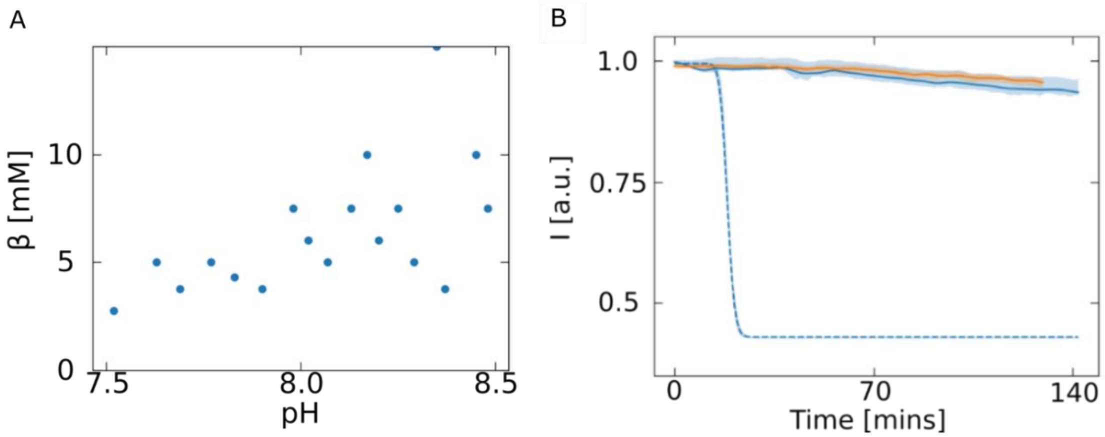

Figure S7. A. Buffer capacity $\beta = \Delta[H+]/\Delta pH$ for 100mM sucrose 10mM Tris 1mM EDTA. Each point represents a single titration at our experimental pH (7.6), indicating that for $\Delta[\mathrm{H}+] = 100$ μM the change of pH is very low $\Delta\mathrm{pH} = 0.02$, implying that H^+ can be exported out of GUVs in amounts comparable to K^+ influx (100s μM) whilst maintaining insignificant pH gradients either side of the GUV membrane. **B.** Stability of pH within Gramicidin A GUVs in response to K^+ transport. We repeated the K^+ transport experiment across GUVs after perfusion of Gramicidin A (5ng/ml), except encapsulating pH sensitive HPTS (10 μM) instead of FAMQ-G4. We display the distribution of HPTS fluorescence within GUVs with (blue) and without (orange) introduction of 1mM K^+ in the external solution (indicated by the reduction in background FAMQ-G4 fluorescence (blue, dashed)).

S10. Measurement of K^+ permeation across electroformed GUVs

We encapsulated FAMQ-G4 (10 μM) within electroformed GUVs (DOPC:DOPG:LR-PE 3:1:0.05) and performed our K^+ transport assay. Figure S6A displays the transported [K^+] over time for both electroformed GUVs (black) and OLA GUVs (red), indicating that K^+ transport significantly faster into OLA GUVs than electroformed GUVs under the same concentration gradients. To compare each membrane system directly we determined distributions of K^+ permeability for GUVs formed by electroformation (black) and OLA (red), displayed in figure S6B.

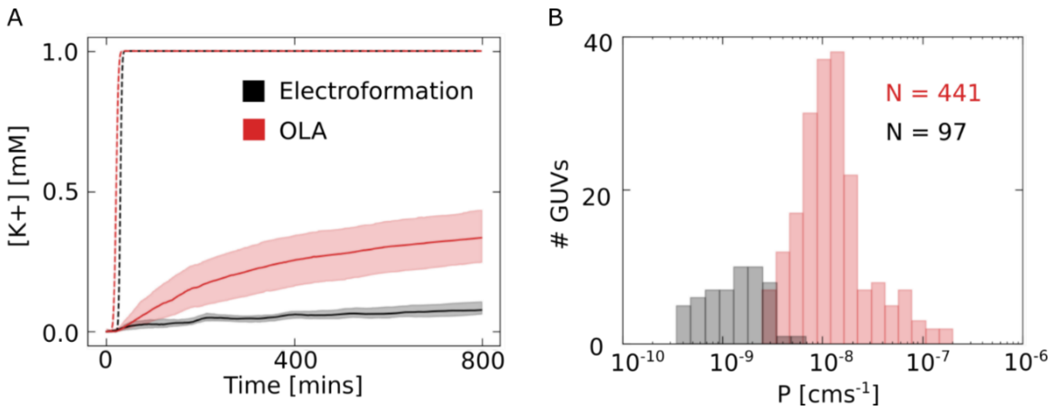

Figure S8. Comparison of K^+ permeability between OLA GUVs (red) and electroformed GUVs (black). **A.** Distributions of lumenal [K^+] development within OLA and electroformed GUVs. At each timepoint the median (solid lines) and lower and upper quartiles of [K^+] (shaded) are shown. In addition, the development of [K^+] outside GUVs is shown as dashed lines colour coded to match the two types of GUVs. **B**. Single GUV distributions of K^+ permeability shown for electroformed (black) and OLA GUVs (red). The sample sizes in each case (N) are indicated.

S11. FAMQ-G4 does not leak from GUVs after perfusion of model ion channels

To confirm the absence of FAMQ-G4 leakage when gramicidin A is reconstituted in the lipid bilayer, we monitored the fluorescence intensity of FAMQ-G4 following gA incorporation (figure S7A). We repeated the same procedure with OmpF to confirm equivalently the absence of significant leakage of FAMQ-G4 (Fig S6 B).

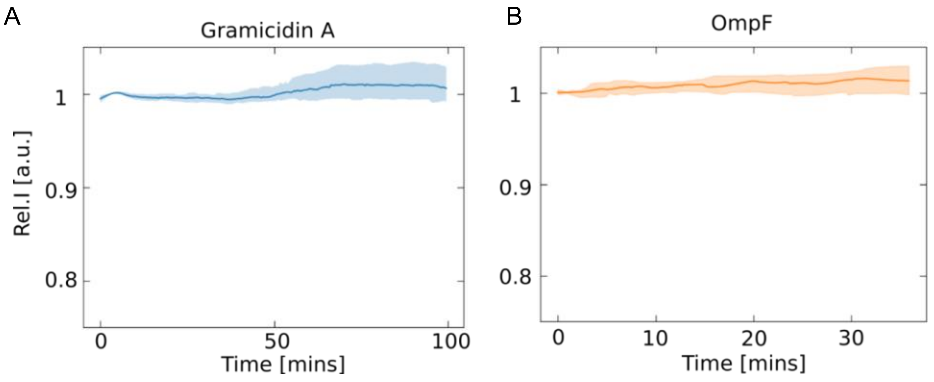

Figure S9. Stability of FAMQ-G4 fluorescence within GUVs after perfusion and washing of 2.6nM gramicidin A **(A)** and 0.012mg/ml OmpF **(B)**. In each case the median fluorescence (solid) and lower and upper quartiles (shaded) are indicated.

S12. Efficiency of gramicidin A incorporation depends on its concentration in solution

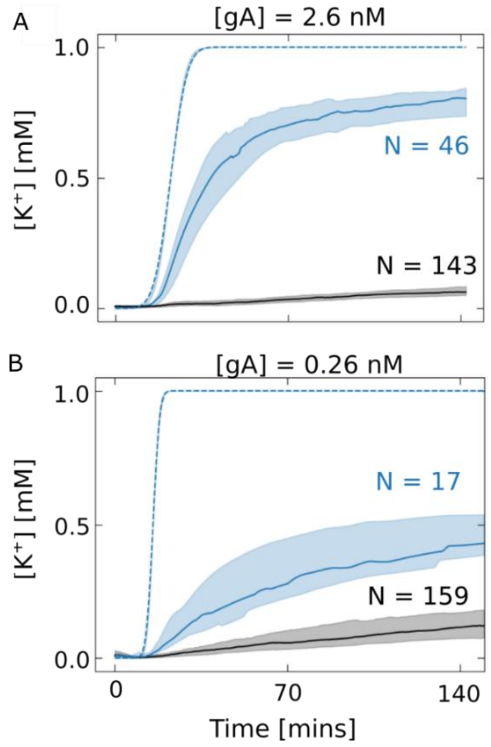

Figure S10. K^+ transport kinetics across GUVs after perfusion of gA at two different concentrations, **A.** [gA] = 2.6nM (5ng/ml) and **B.** [gA] = 0.26nM (0.5ng/ml)). In each experiment we identified two populations of transport rates – one which varied with [gA] (blue) and the other which did not vary significantly (black), indicating heterogeneous distributions of pore activity amongst GUV populations. The temporal variation of lumenal and external (dashed lines) K^+ concentrations is indicated by the solid and dashed lines, respectively.

S13. K^+ transport across OmpF containing GUVs

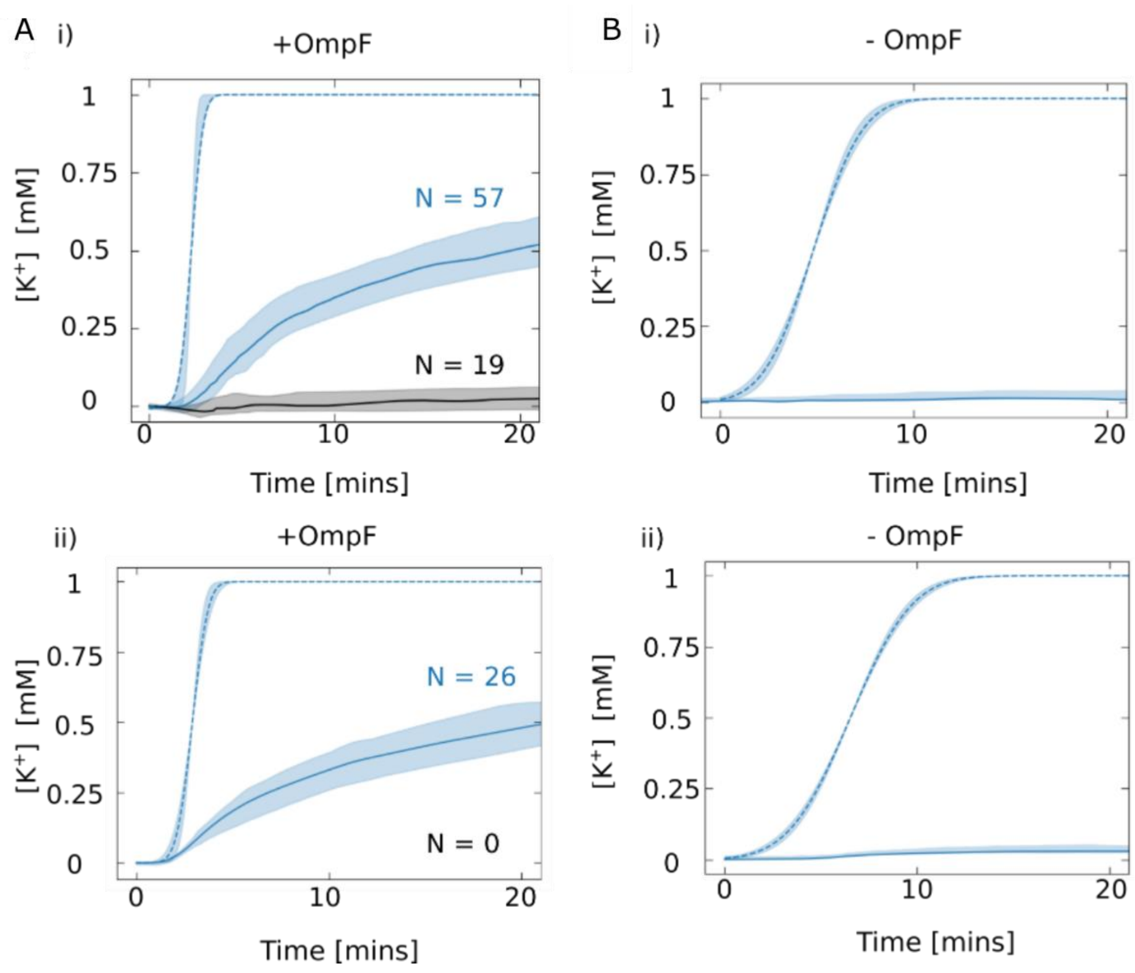

Figure S11. A. K^+ transport kinetics for GUVs after perfusion of OmpF repeated twice with the same perfusion concentration (i and ii). In one experiment we identified two populations of transport rates, one which was significantly faster (blue) than the other (black) which we identified as OmpF containing GUVs. **B.** Control measurement after repeating the experimental procedure without OmpF but including the same concentration of OPOE (see Experimental Section).

S14. Microfluidic device CAD Designs

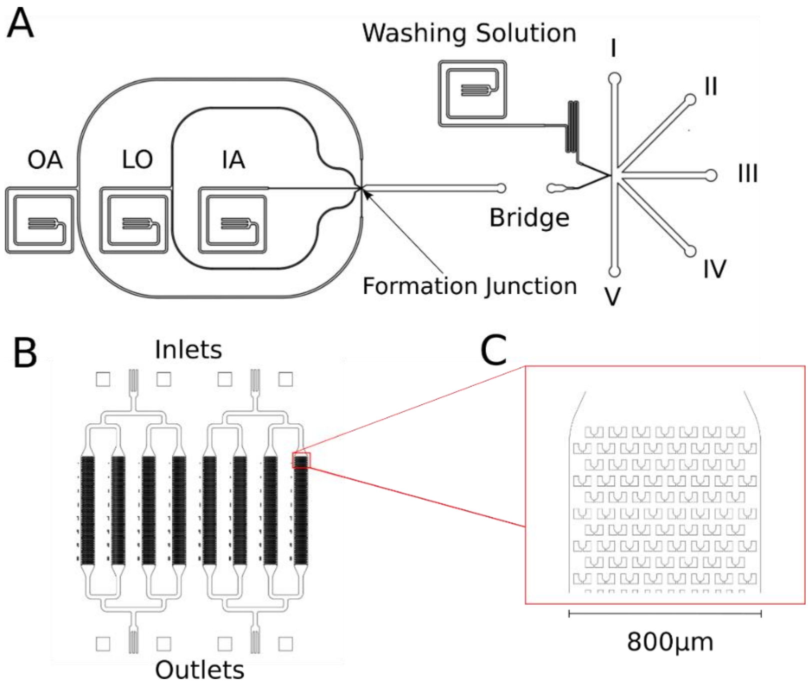

Figure S12. A. CAD for the integrated microfluidic GUV production and purification device as described previously[1]. **B.** CAD for the microfluidic hydrodynamic trapping device, showing individual traps (red box).

S15. K^+ flux density approximately equals H^+ counter flux during linear regime

In the context of our experiment, membrane potential development Δψ is proportional to the excess concentration of ions, $n,$ within the GUVs as a result of K^+ transport. As Cl^- flux through cation-selective channels such as OmpF and Gramicidin A is negligible under our experimental conditions[3-5], $n = [K^+]_{int} - [H^+]_{export}$, and therefore $[H^+]_{export} = [K^+]_{int} - n.$ We may show n is small during the linear regime ($\Delta\psi < \frac{RT}{F} \approx$ 25mV at 25°C), using the relation $\Delta\psi = \frac{Frn}{3c}$, where F is the Faraday constant, r the GUV radius (r = 10 μm) and c is the specific capacitance of the lipid bilayer (c = 1 $\mu\mathrm{Fcm}^{-2}$). Solving the equation for Δψ < 25mV we obtain that $n < 0.8\ \mu$M. For transport of K^+ across gA (see figure 4C in the main text), the linear regime extends up $[K^+]_{int} \sim 100\ \mu$M. Therefore $[H^+]_{export} \sim [K^+]_{int}$ to within ca. 1%. When Δψ is lower earlier on in the transport process, n is even smaller, and so it can be concluded that potassium influx and proton efflux are similar ($J_{K^+} \sim J_{H^+}$) within the linear regime.

References

1. Tivony, R.; Fletcher, M.; Keyser, U. F., Quantifying proton-induced membrane polarization in single biomimetic giant vesicles. *Biophysical Journal* **2022**. DOI: https://doi.org/10.1016/j.bpj.2022.05.041
2. Morzy, D.; Rubio-Sánchez, R.; Joshi, H.; Aksimentiev, A.; Di Michele, L.; Keyser, U. F., Cations Regulate Membrane Attachment and Functionality of DNA Nanostructures. *Journal of the American Chemical Society* **2021,** *143* (19), 7358-7367.
3. Alcaraz, A.; Nestorovich, E. M.; Aguilella-Arzo, M.; Aguilella, V. M.; Bezrukov, S. M., Salting Out the Ionic Selectivity of a Wide Channel: The Asymmetry of OmpF. *Biophysical Journal* **2004,** *87* (2), 943-957.
4. Im, W.; Roux, B. t., Ion Permeation and Selectivity of OmpF Porin: A Theoretical Study Based on Molecular Dynamics, Brownian Dynamics, and Continuum Electrodiffusion Theory. *Journal of Molecular Biology* **2002,** *322* (4), 851-869.
5. Finkelstein, A.; Andersen, O. S., The gramicidin a channel: A review of its permeability characteristics with special reference to the single-file aspect of transport. *Journal of Membrane Biology* **1981,** *59* (3), 155-171.
